# Functional Outcomes Following a 10-Week PD Warrior Program: A Real-World Clinical Follow-Up of a Neuroplasticity-Driven Exercise Approach

**DOI:** 10.3390/jcm15207915

**Published:** 2026-10-14

**Authors:** Orla Hares, Melissa McConaghy, Victoria D’Alessio, Ann Liebert

**Affiliations:** 1 School of Rehabilitation Sciences, McMaster University, Hamilton, ON L8S 4L8, Canada; orla@gaitwayneurophysio.com; 2 Gaitway Neurophysio, Hamilton, ON L8P 2B6, Canada; research@gaitwayneurophysio.com; 3 Macquarie University, Sydney 2109, Australia; melissa@pdwarrior.com; 4 Kolling Institute, Sydney University, Sydney 2064, Australia; 5 College of Business, Recreation and Education, Shepherd University, Shepherdstown, WV 25443, USA

**Keywords:** Parkinson’s disease, exercise, PD Warrior, functional mobility

## Abstract

**Background/Objectives:** Parkinson’s disease (PD) is the fastest-growing neurodegenerative condition worldwide, placing increasing burden on individuals, families, and healthcare systems. While pharmacological and device-assisted therapies remain central to management, their effectiveness may diminish over time and can be accompanied by significant adverse effects. Targeted, high-intensity exercise has emerged as an important adjunctive therapy, with the PD Warrior program representing a structured, neuroplasticity-driven approach designed specifically for people with PD. To evaluate functional mobility, gait, balance, and disease stage outcomes following participation in a 10-week PD Warrior program delivered within a physiotherapy clinic as part of a multimodal treatment approach. **Methods:** 29 participants with idiopathic Parkinson’s disease (eight females, 21 males; Hoehn & Yahr Stages 1–3) had follow-up assessments at various times after a 10-week PD Warrior program. The program consisted of one weekly 60 min high-intensity, neuroplasticity-based group class combined with a structured home exercise program performed 5–6 days per week. Outcomes included the Timed Up-and-Go test (TUG), 10-Meter Walk Test (10MWT; measuring time and steps), Mini-Balance Evaluation Systems test (Mini-BESTest), and Hoehn & Yahr (H&Y) Stage. Pre- and post-program data were analyzed using non-parametric statistics and the TUG and mini-BEST tests were interpreted with reference to published minimal clinically important difference (MCID) thresholds. **Results:** Both median TUG time and median step length showed a statistical improvement after the PD Warrior program. 13 of 24 participants showed an improvement in TUG above MCID with six showing a deterioration. Similarly, with step length, 12 of 20 participants showed improvement and five showed a deterioration. There was a nonsignificant increase in median walking speed, with 14 of 21 participants showing an improvement and seven showing a deterioration. For balance, a similar number of participants showed improvement (12) and deterioration (10), while for H&Y disease Stage, most participants (18 of 29) remained the same, although slightly more improved (eight) than deteriorated (three). **Conclusions:** Participation in a 10-week PD Warrior program was associated with improvements in functional mobility and gait efficiency, but not balance, while disease stage was maintained. While these findings are consistent with the goals of PD Warrior as a structured, neuroplasticity-driven exercise intervention within a multimodal model of Parkinson’s disease care, more work is needed to establish any link between the intervention and improvements in functional mobility.

## 1. Introduction

Parkinson’s disease (PD) is the fastest-growing neurodegenerative condition globally, with prevalence expected to double over the coming decades to over 12 million by 2040 as populations age [1]. The progressive nature of the disease places substantial and growing demands on individuals, caregivers, and healthcare systems worldwide.

Pharmacological and device-assisted therapies, including deep brain stimulation, remain essential components of PD management; however, long-term treatment is often complicated by diminishing therapeutic response, medication side effects, and increased symptom complexity. Consequently, there is increasing interest in non-pharmacological interventions that not only address motor impairment but also promote self-management and sustained behavioral change.

A number of non-pharmaceutical therapies to manage the clinical signs and symptoms of PD are currently in use clinically or are under investigation, most often as complimentary treatments alongside standard pharmaceutical therapy. There is some evidence for the effectiveness of acupuncture [2], dietary changes [3], fecal microbiome transplantation [4], bright light therapy [5], physical therapy [6], and photobiomodulation [7] to manage symptoms. However, the intervention with the most evidence is exercise, which has long been recognized as beneficial for people with PD [8]. General exercise and higher physical activity can slow the deterioration in postural stability and gait and reduce the clinical progression of PD [9]. While it appears that no matter the level of activity or the stage of the disease, there are benefits from regular exercise [10], there is growing evidence supporting the particular effectiveness of high-intensity exercise for Parkinson’s symptoms [10,11], including aerobic exercise [12] and high-intensity treadmill [13]. Parkinson’s specific exercise programs that are cognitively engaging may also drive neuroplastic change [14], reduce neuroinflammation, and optimize the effect of dopamine [15]. The PD Warrior program is distinct in its integration of high-effort, amplitude-based movement training, cognitive challenge, and task-specific cueing, alongside a structured behavioral framework designed to support adherence and self-efficacy. This approach aims to address key pathophysiological features of Parkinson’s disease, including reduced movement amplitude, impaired automaticity, and diminished motor drive, while promoting long-term engagement in exercise. While emerging studies on the effect of intensive exercise would support the effectiveness of the PD Warrior program, there have been few studies that have specifically assessed the efficacy of the program. There remains a need for real-world clinical data examining outcomes of PD Warrior when embedded within routine physiotherapy practice.

The purpose of this retrospective clinical audit was to evaluate functional mobility, gait, balance, and disease staging outcomes following participation in a 10-week PD Warrior program delivered in a physiotherapy clinic, as part of a broader multimodal model of care for people with Parkinson’s disease.

## 2. Methods

The outcome data were collected as part of routine clinical care, and the audit did not involve prospective participant recruitment, assignment of an intervention or research-specific procedures and therefore fell outside the scope of Research Ethics Board (REB) review (https://ethics.gc.ca/eng/policy-politique_tcps2-eptc2_2022.html; last accessed on 23 September 2026).

**Figure 1:**
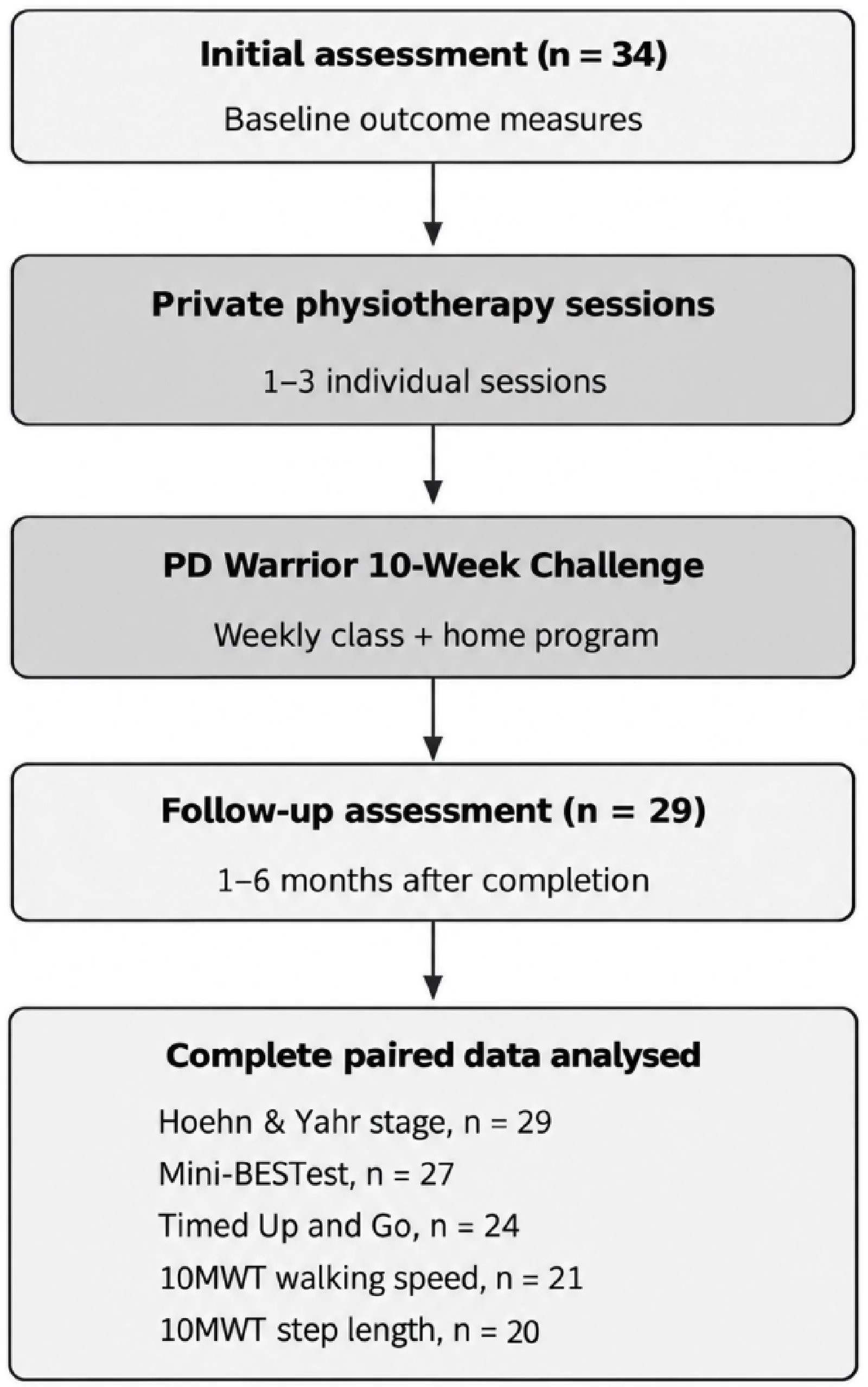
Experimental design.

This was a retrospective clinical audit of follow-up assessments of 29 real-world participants who had completed the PD Warrior 10-Week Challenge through the Parkinson’s Wellness Innovation Centre in Hamilton, Canada (Figure 1). Inclusion criteria consisted of a diagnosis of idiopathic Parkinson’s disease by an independent neurologist, completion of the 10-week PD Warrior Challenge program and clinical assessments available for before and after the program. The cohort included individuals newly diagnosed with PD as well as those up to 7 years post-diagnosis. Medication status and disease duration were not reported.

The 10-Week Challenge is a comprehensive, multi-modal intervention that incorporates structured education, coaching, and motivational support to promote adherence, self-efficacy, and sustained behavioral change. Participants in this study engaged in in-person, group-based classes where they followed instructor-led demonstrations and received real-time feedback. The program includes 10–18 individualized exercises that focus on key physical symptoms (especially bradykinesia, tremor and agility/mobility). Participants received an initial in-person assessment and were individually coached to perform the program’s core set of 10 high-effort exercises, with personalized cues to help them consistently reach 80% of exertion, using the PD Warrior Rating of Perceived Exertion (RPE) scale (Supplementary Material S1). Progression in the program is individualized, based on ability and clinical presentation. They also accessed the PD Warrior online support platform, which provided structured guidance, weekly themes, and tools to support adherence and self-efficacy beyond the program. Participants were encouraged to complete a structured home exercise program 5–6 days per week, reinforcing core program principles such as repetition, movement amplitude, balance challenge, agility, and cognitive engagement. However, due to the retrospective nature of the audit, adherence to the home exercise program and changes in concurrent therapies, such as participation in concurrent exercise programs, were not systematically recorded.

Assessment of outcomes was conducted as part of the typical follow-up of clients in the center and timing was influenced by factors such as individual clinical needs, ongoing participation in services, appointment scheduling, and patient availability. Assessments were administered by two registered physiotherapists experienced in the assessment and treatment of Parkinson’s disease symptoms. Participants were assessed during their medication “on” phase. Outcome measures consisted of Hoehn and Yahr (H&Y) staging, Mini-Balance Evaluation Systems test (Mini-BESTest), Timed Up-and-Go (TUG), and 10-Meter Walk Test (10MWT), all performed using standardized protocols (Supplementary Material S2). These measures were selected to assess disease severity, dynamic balance, functional mobility, and gait performance. Participant data was excluded for individual outcomes if either pre- or post-assessment was unavailable.

All statistical tests were performed as an exploratory analysis by a researcher external to the study. Normality of data was assessed using the Shapiro–Wilk test. Given the presence of non-normal distributions and small sample sizes, descriptive statistics are presented as median and interquartile range (IQR), and pre- and post-program outcomes were analyzed using Wilcoxon signed-rank tests. The relationship between the change in outcome measures and length of time since completing the 10-week challenge was tested using Spearman’s rank correlation coefficient. Statistical significance was set at *p* < 0.05; however, it is important to note that statistical and clinical significance are independent.

Clinical meaningfulness for the TUG was evaluated using the minimal clinically important difference (MCID) threshold [16] of 1.14 s, with participants classified as demonstrating improvement, improvement above MCID, stability, and deterioration. For the Mini-BESTest, a change of greater than 4 points was defined as the MCID [17].

## 3. Results

Assessment of outcomes is summarized in Table 1, with a complete participant outcome data provided in Appendix A. No adverse events were recorded.

**Figure 2:**
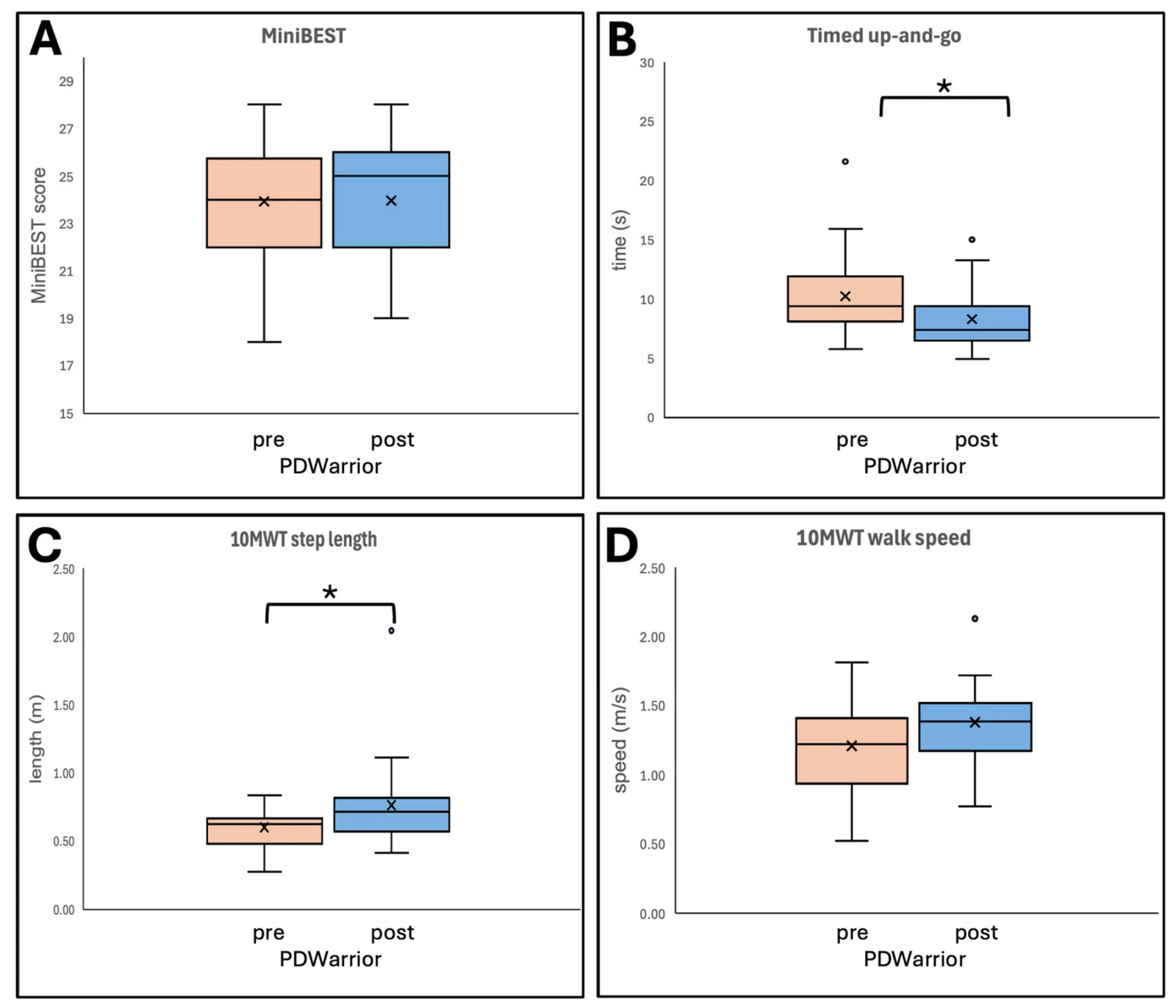
Boxplots showing pre- and post-intervention performance. (**A**) Mini-BESTest score, (**B**) Timed Up-and-Go, (**C**) 10-Meter Walk Test (10MWT) step length, and (**D**) 10MWT walking speed. Significant improvements were observed in TUG and step length following the intervention (* *p* < 0.05).

Data sets were not normally distributed as determined by the Shapiro–Wilk test, therefore change after the PD Warrior 10-week challenge was assessed using the Paired Wilcoxon signed-rank test. There was a significant improvement in TUG times after the PD Warrior intervention (Figure 2B), with 16 of 24 participants showing an improvement, 13 of whom showed an improvement above MCID (Table 1). Six of 24 participants showed a deterioration in TUG. There was also a significant improvement in step length in the 10MWT (Figure 2C) with 12 of 20 participants needing fewer steps to complete the test (Table 1) and only five participants deteriorated in step length. There was a small but non-significant improvement in walking speed during the 10MWT (Figure 2D) with 14 of 21 participants showing an improvement, and seven participants deteriorating in speed. Approximately equal numbers of participants improved (12) or deteriorated (10) in their balance, with none improving above the MCID threshold (Figure 2A).

There was no significant relationship, as assessed by Spearman’s rank correlation coefficient, between the timing of the follow-up assessment and the change in any outcome measure (Appendix A).

## 4. Discussion

This retrospective clinical audit demonstrated that participation in a PD Warrior 10-week program delivered in a real-world physiotherapy setting was associated with clinically meaningful improvements in functional mobility and gait efficiency in individuals with mild to moderate Parkinson’s disease. Due to the design of the study, no inferences regarding a causal link can be drawn. However, the significant improvements in TUG performance and step length of the cohort, together with the high number of MCID improvements for the TUG warrant further studies to determine if a causal link exists. If linked to the PD Warrior intervention, meaningful changes in mobility would likely translate into enhanced day-to-day function for people with Parkinson’s disease and would be consistent with the program’s emphasis on movement amplitude and functional task execution. In contrast there was less overall improvement in balance as measured by the Mini-BESTest. Interestingly, three of four participants who were H&Y Stage 3 showed a deterioration in balance, despite two of them improving their H&Y Stage. Since balance instability is characteristic in Stage 3, this result is worthy of further exploration. 

Most participants did not change their disease stage during the study. Despite statistically significant improvements in some measures, variability in individual responses was observed across all outcomes. Given the progressive nature of Parkinson’s disease, the maintenance of function would itself represent a clinically important outcome if future studies could demonstrate a link between the program and changes in symptoms. Future studies may also benefit from incorporating additional measures of dynamic stability, dual-task performance, and reactive balance.

These findings are consistent with the growing body of evidence demonstrating the benefits of exercise in Parkinson’s disease, particularly when delivered at sufficient intensity and frequency [18]. Higher levels of physical activity have been associated with a more favorable clinical trajectory [9]. Specific exercise regimens, such as resistance training [11], aerobic exercise [12], and high-intensity treadmill [13] have been shown to have a positive effect on the clinical motor signs of PD, including the motor component (III) of the MDS-UPDRS score. The PD Warrior exercise program, in a study of 17 participants, was shown to significantly improve motor outcomes, including the MDS-UPDRS-III (motor) scores, walking times and TUG dual-task times [19].

Animal studies have suggested that moderate to high-intensity exercise not only improved motor signs of PD, but was also neuroprotective [20], upregulating both BDNF and GDNF [21], increasing striatal dopamine release [22] and improving dopamine binding to dopamine receptors [23]. Exercise in animal models of PD has also been shown to reverse substantia nigra vascularization decline and increase progenitor cell proliferation and migration [24] as well as improving neuroplasticity [25]. While there are many caveats when generalizing the effects of any treatment on animals to humans, several clinical studies have suggested that neuromodulation factors, such as BDNF [26], are increased with exercise and may have a role in neuroprotection. A recent imaging study has also shown that intense exercise can increase dopamine transporter availability and neuromelanin levels, suggesting a neuroprotective effect of exercise [27]. Future studies of the effect of PD Warrior on motor symptoms would benefit from the inclusion of markers and/or the imaging of neuroprotection.

The very small number of participants that showed deterioration in TUG performance is particularly notable given the progressive trajectory of Parkinson’s disease. Improvements in gait efficiency, reflected by increased step length over 10 m, suggest positive adaptations in motor control that are likely to translate into everyday functional mobility. People with Parkinson’s disease are also known to have a shorter step length and a slower walk speed than healthy adults of the same age [28], and these are both independently associated with falls risk [29]. If it can be shown that the PD Warrior intervention can lower the risk of falling, this would greatly improve quality of life by reducing injury and pain, maintaining independence, and alleviating the fear of falling that can lead to avoidance or restriction of exercise [30], which is counterproductive to the need for physical activity.

The PD Warrior program is distinct from general exercise approaches in that it integrates high-effort motor training with task-specific cueing, cognitive engagement, and a structured behavior-change framework. This combination is designed to target key mechanisms underpinning motor impairment in Parkinson’s disease, including reduced movement amplitude, impaired automaticity, and decreased motor drive. While the improvements observed in step length and functional mobility align closely with these mechanistic targets and support the theoretical basis of the program, more study is needed to establish the efficacy of the program.

A notable feature of the PD Warrior approach is its emphasis on behavioral adherence and self-directed practice. Participants were supported to perform high-effort exercise multiple times per week, with coaching, education, and structured progression embedded throughout the program. This model extends beyond supervised exercise sessions and may contribute to sustained engagement and continued practice beyond the formal intervention period. While adherence to the home program was not formally quantified in this audit, this behavioral component is likely to have influenced the outcomes observed, particularly given the variability in the time between the completion of the program and follow-up assessment.

Importantly, PD Warrior represents more than an exercise prescription alone. Its integration of structured progression, cognitive challenge, and sustained behavioral support may underpin the functional changes observed and differentiate it from non-specific exercise approaches. When embedded within a multimodal model of care, this approach offers a scalable and clinically feasible framework to support long-term self-management and engagement in physical activity for people living with Parkinson’s disease.

The findings of this audit must be interpreted in the context of a number of limitations. The retrospective design and absence of a control group preclude causal inference, and the observed improvements cannot be attributed solely to the PD Warrior program. The retrospective design also introduces the possibility of both selection and information bias. However, the analysis was based on predefined clinical outcome measures and established statistical procedures, and the use of two trained assessors helped support consistent scoring of the outcome measures and reduce reliance on a single assessor. Participants were included based on completion of the PD Warrior 10-Week Challenge and availability of routinely collected clinical assessment data rather than on the magnitude or direction of their outcomes. Data was extracted and verified by one author (OH) and while no external researcher reviewed the dataset, statistical analysis was conducted by an independent researcher. Furthermore, reliance on clinical records resulted in missing data for some outcome measures and limited control over assessment timing and other potentially relevant variables. The cohort was also very heterogeneous, ranging from H&Y Stage 1 to 3 and from newly diagnose to 7 years post-diagnosis, which would have influenced any improvement seen, particularly balance which is a defining feature of H&Y stages. Participants were also engaged in a broader multimodal care environment, which may have contributed to changes in outcome measures. Participants were not monitored for their adherence to the PD Warrior after the 10-week program, and differences in exercise regimens, concurrent therapies, medication changes, and ongoing exercise would affect outcomes. There was also considerable variability in the timing of follow-up assessments. Additionally, missing data across outcome measures limit the precision of the findings. Despite these limitations, the audit provides valuable real-world insight into the implementation and potential impact of a structured, neuroplasticity-driven exercise program in routine clinical practice.

## 5. Conclusions

In this retrospective clinical audit, improvements in a number of clinical motor signs, including TUG and walking speed and step length, and they were observed in participants who attended a 10-week PD Warrior program, delivered in a real-world physiotherapy clinic setting. This is consistent with the program’s emphasis on high-effort, task-specific training targeting movement amplitude, motor control, and behavioral engagement. Despite the limitations of variable timing in follow-up assessments, some missing data, absence of a control group and a heterogenous cohort, this audit provides encouraging real-world evidence that a targeted, neuroplasticity-driven exercise approach can be effectively implemented in clinical practice. The observed improvements in key functional outcomes, particularly those aligned with PD Warrior’s core training principles, support further investigation in more rigorously controlled studies.

## Figures and Tables

**Table 1:** Summary of outcome measures before and after the 10-week challenge.

Outcome Measure	Pre-Program Median (IQR)	Post-Program Median (IQR)	Improved n/N (Above MCID)	Deteriorated n/N	W (*p*)
H&Y Stage	2 (1)	1 (1)	8/29	3/29	22 (0.308)
Mini-BESTest score	24 (3.00)	25 (4.00)	12/27 (0)	10/27	120 (0.844)
TUG time (s)	9.2 (3.54)	7.4 (2.73)	16/24 (13)	6/24	45 (0.007) *
10MWT step length (m)	0.63 (0.18)	0.71 (0.24)	12/20	5/20	26.5 (0.019) *
10MWT speed (m/s)	1.23 (0.54)	1.39 (034)	14/21	7/21	67 (0.096)

H&Y = Hoehn and Yahr stage; Mini-BESTest = Mini-Balance Evaluation Systems test; TUG = Timed Up-and-Go; 10MWT = 10-Meter Walk Test; n = number of participants showing improvement or deterioration, N = the total number of participants with complete data available for that outcome measure; MCID = number of participants showing change beyond the minimal clinically important difference threshold; W = Wilcoxon signed-rank test statistic; *p* = *p*-value; * = significant *p* value (*p* < 0.05).

## Data Availability

The original contributions presented in this study are included in the article/Supplementary Material. Further inquiries can be directed to the corresponding author.

## Supplementary Materials

The following supporting information can be downloaded at https://www.mdpi.com/article/10.3390/jcm15207915/s1, S1: PD Warrior Assessment Sheet; S2: Outcome Measure Testing Protocols.

## Abbreviations

The following abbreviations are used in this manuscript:

10MWT	10-Meter Walk Test
H&Y	Hoehn and Yahr
IQR	Inter-quartile range
Mini-BESTest	Mini-Balance Evaluation Systems test
MCID	Minimal clinically important difference
MDS-UPDRS	Movement Disorder Society Unified Parkinson’s disease rating scale
PD	Parkinson’s disease
RPE	Rating of Perceived Exertion
TUG	Timed up-and-go

## Appendix A. Individual Participant Outcomes

**Table A1:** Results for individual participant outcome measures and statistical summary.

Participant	Sex	Age	Weeks b/n 10-Week Challenge & Follow-Up Assessment	H&Y Stage		Mini-BESTest Score		TUG Time (s)		10MWT Step (m)		10MWT Speed (m/s)	
				Pre	Post	Pre	Post	Pre	Post	Pre	Post	Pre	Post
1	F	74	43	1	-	26	24↓	21.6	10.6↑*	—	0.63	—	0.59
2	M	73	51	1	1	24	25↑	10.6	7.5↑*	0.50	0.56↑	0.94	0.91↓
3	M	81	34	2	2	18	14↓	11.5	13.3↓	—	—	—	—
4	F	79	10	3	2↑	23	19↓	—	—	—	—	—	—
5	M	76	34	3	3	20	19↓	9.1	15↓	0.56	0.56	1.23	0.96↓
6	F	66	38	3	1↑	23	20↓	—	12.5	0.28	0.71↑	0.52	1.34↑
7	M	72	45	1	2↓	28	28	9.6	11.4↓	0.67	0.53↓	1.71	1.32↓
8	M	73	10	1	2↓	22	22	13.2	—	0.48	0.42↓	0.76	0.87↑
10	M	66	63	1	1	26	25↓	7.9	6.7↑*	0.67	0.77↑	1.20	1.50↑
11	F	70	55	1	2↓	19	21↑	14.4	11.1↑	0.67	0.83↑	1.25	1.47↑
12	M	65	35	2	2	23	24↑	—	—	0.45	0.71↑	0.90	1.53↑
13	F	70	43	1	1	22	23↑	11.6	5.8↑*	0.48	0.67↑	0.93	1.39↑
14	F	39	9	2	2	-	26	—	5.8	—	0.67	—	1.37
15	M	57	42	1	1	25	26↑	11.6	8.9↑*	—	0.83	—	1.47
17	M	67	25	2	2	25	21↓	12.9	8.6↑*	0.71	1.11↑	1.28	2.13↑
18	M	63	20	1	1	27	28↑	7.7	6.5↑*	0.67	0.83↑	1.25	1.47↑
19	M	70	21	2	1↑	28	28	8.6	4.9↑*	0.63	2.04↑	1.34	0.77↓
20	M	62	19	3	2↑	22	25↑	15.9	9.4↑*	0.50	0.71↑	0.71	1.49↑
21	M	63	17	1	1	25	27↑	7.0	7.0	—	—	—	—
22	M	71	8	1	1	—	26	8.2	8.7↓	—	0.63	1.27	1.16↓
23	M	64	9	1	1	25	24↓	8.4	7.3↑	0.83	0.63↓	1.61	1.27↓
24	F	69	8	2	1↑	24	25↑	9.8	7.5↑*	—	0.59	—	1.14
25	M	54	10	2	2	22	22	9.2	6.4↑*	—	1.11	—	1.73
28	M	59	7	1	1	27	24↓	5.8	6.5↓	0.63	0.77↑	1.72	1.66↓
30	M	78	9	2	1↑	25	25	8.8	7.1↑*	0.67	0.63↓	1.15	1.20↑
31	M	60	9	2	1↑	24	26↑	8.2	7.2↑	0.56	0.56	1.10	1.18↑
32	M	69	9	1	1	28	27↓	6.3	6.3	0.83	0.83	1.82	2.15↑
33	M	77	9	2	1↑	22	25↑	10.1	7.4↑*	0.48	0.67↑	0.93	1.42↑
34	F	61	9	2	2	23	25↑	6.4	7.2↓	0.83	0.71↓	1.71	1.72↑
Median		69	19	2	1	24	25	9.2	7.4	0.63	0.71	1.23	1.39
IQR		10.3	31.0	1	1	3.00	4.00	3.54	2.73	0.18	0.24	0.54	0.34
Wilcoxon W				22 *p* = 0.308		120 *p* = 0.844		45 *p* = 0.007 **		26.5 *p* = 0.019 **		67 *p* = 0.096	
Median difference (95% CI)				0 (–1, 0.5)		0 (–1, 1)		–2.01 (–3.06, 0.94)		0.14 (0.03, 0.25)		0.16 (–0.03, 0.37)	
Effect size				0.31		0.04		0.58		0.57		0.36	
number improved				8/29		12/27		16/24 (13↑*)		12/20		14/21	
number deteriorated				3/29		10/27		6/24		5/20		7/21	
Spearman’s r_s_ (n)						–0.068 (27) *p* = 0.74		–0.315 (24) *p* = 0.13		0.320 (21) *p* = 0.16		0.214 (21) *p* = 0.35	

H&Y = Hoehn and Yahr stage; Mini-BESTest = Mini-Balance Evaluation Systems Test; TUG = Timed Up and Go; 10MWT = 10-Meter Walk Test; IQR = interquartile range; W = Wilcoxon signed-rank test statistic; CI = confidence interval; n = number of participants; ↑ = improved after intervention; ↓ = declined after intervention; *p* = *p*-value; * = improvement beyond minimal clinically important difference threshold; ** = significant *p* value (*p* ≤ 0.05).
