# Supplementary material for: Functional Outcomes Following a 10-Week PD Warrior Program: A Real-World Clinical Follow-Up of a Neuroplasticity-Driven Exercise Approach
Source: J Clin Med. 2026 Oct 14;15(20):7915. doi: 10.3390/jcm15207915 (PMC13706282; doi:10.3390/jcm15207915)
Supplement: Supplementary file 1 [file jcm-15-07915-s001.zip › Supplementary material S1 .pdf]

PD ASSESSMENT SHEET

Name________________________________**DOB**______________

Therapist_______________________**Time**________**ON/OFF**______

WHAT DO YOU WANT TO ACHIEVE FROM THERAPY?

1.____

2.____

3.____

WHAT ARE YOUR MAIN PROBLEMS?

1.____

2.____

3.____

CURRENT HISTORY

- When were you diagnosed with Parkinson's disease?__________________________
- Do you have a specialist?____
- Do you see other health professionals?_____________________________________

MEDICAL HISTORY

- Do you have any other medical conditions that you see the doctor about? ◯ Yes ◯ No
- Do you have a history of cardiac problems including high blood pressure? ◯ Yes ◯ No
- Do you have a history of respiratory problems including breathlessness? ◯ Yes ◯ No
- Do you have any other neurological conditions? ◯ Yes ◯ No
- Do you have Diabetes / Epilepsy / infectious condition ◯ Yes ◯ No
- Do you suffer from incontinence? ◯ Yes ◯ No
- Have you fallen in the past 12 months? ◯ Yes ◯ No
- Have you experienced freezing of gait in the past month? ◯ Yes ◯ No
- Have you had a recent change in medication? ◯ Yes ◯ No
- Have you had a recent injury, pain, any surgery or illness? ◯ Yes ◯ No
- Do you suffer from Anxiety / lack of motivation / depression ◯ Yes ◯ No

SOCIAL HISTORY

- Do you live alone?____ ◯ Yes ◯ No
- Are you working?____ ◯ Yes ◯ No
- Are you managing everyday domestic chores? ◯ Yes ◯ No
- What transportation do you use? Drive / Public Transport / Taxi / Passenger / Other
- What other activities/hobbies do you enjoy?_______________________________________

Comments____

WHAT EXERCISE DO YOU DO?	WHAT ARE YOUR MEDICATIONS?

MAIN MOTOR SYMPTOMS (circle)			MAIN NON-MOTOR SYMPTOMS		
Slowness	Tremor	Freezing	Memory	Anxiety	Mood
Balance	Voice	Post DBS	Sleep	Fatigue	Bladder
Dyskinesia	Dystonia	other	Bowel	Cramps	other

TREATMENT PLAN & SUMMARY: (THERAPIST SIGNATURE)______________________________

ASSESSMENT MEASURES

	PRE<br>Week 0		POST<br>Week 10- 12		FOLLOW-UP<br>Week 26	
**Date**						
**Ax Time**						
	**Secs**	**Steps**	**Secs**	**Steps**	**Secs**	**Steps**
**10mwt**						
**10mwt (MOTOR)**						
**10mwt (COGNITIVE)**						
**10 Bean test/9 Hole peg Test**						
**PDQ-39**						
**SPDDS**						
**MiniBEST**						
**Activity log**						
**Challenge task**						
	**Metres**	**RPE**	**Metres**	**RPE**	**Metres**	**RPE**
**6mwt**						
**Observation gait/other**						

PD WARRIOR RPE SCALE

10%	I’m not even pretending to do this exercise
20%	I’m doing the exercise with insignificant effort
30%	I’m going through the motions and feel less than impressive
40%	I could do this exercise all day
50%	I could do this exercise at least fifty times and not break a sweat
60%	I am starting to generate power and force in my movements
70%	I am starting to feel athletic and definitely breaking a sweat
**80%**	**I am powerful and feisty-**<br>**I am moving like a PD Warrior!**
90%	I am at risk of levitating I am moving so vigorously
100%	I am going to hurt myself, someone next to me, or the furniture

10 METRE WALK TEST

General Information:

Individual walks without assistance 14 meters and the time is measured for the intermediate 10 meters to allow for acceleration and deceleration.

- start timing when the toes of the leading foot crosses the 2-meter mark
- stop timing when the toes of the leading foot crosses the 10-meter mark
- assistive devices can be used but should be kept consistent and documented from test to test
- if physical assistance is required to walk, this should not be performed
- can be performed at preferred walking speed or fastest speed possible
- documentation should include the speed tested (preferred vs. fast)
- collect three trials and calculate the average of the three trials

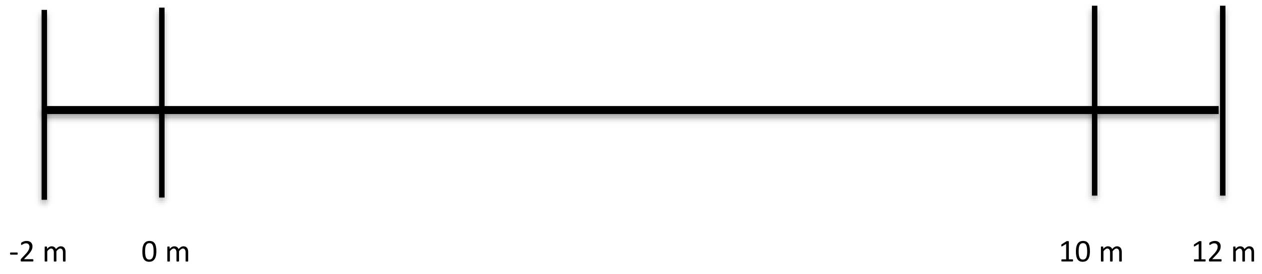

Set-up:

- measure and mark a 14-meter walkway
- add a mark at 2-meters
- add a mark at 10-meters

Patient Instructions:

Normal comfortable speed: "I will say ready, set, go. When I say go, walk at your normal comfortable speed until I say stop"

Additional motor task: "After you start walking at your normal speed, please click the clicker on the right hand, transfer to the left hand and click again. Transfer back to the right hand and repeat until I say stop."

Additional cognitive task: "After you start walking at your normal speed, please start to spell your full name backwards".

PD WARRIOR

10 METRE WALK TEST

VALUES

METRES	SECS	M/S	STEPS	STRIDE CM
10	35	0.29	35	57.14
10	30	0.33	30	66.67
10	25	0.40	25	80.00
10	20	0.50	20	100.00
10	18	0.56	18	111.11
10	16	0.63	16	125.00
10	14	0.71	14	142.86
10	12	0.83	12	166.67
10	10	1.00	10	200.00
10	9	1.11	9	222.22
10	8	1.25	8	250.00
10	7	1.43	7	285.71
10	6	1.67	6	333.33
10	5	2.00	5	400.00
10	4	2.50	4	500.00

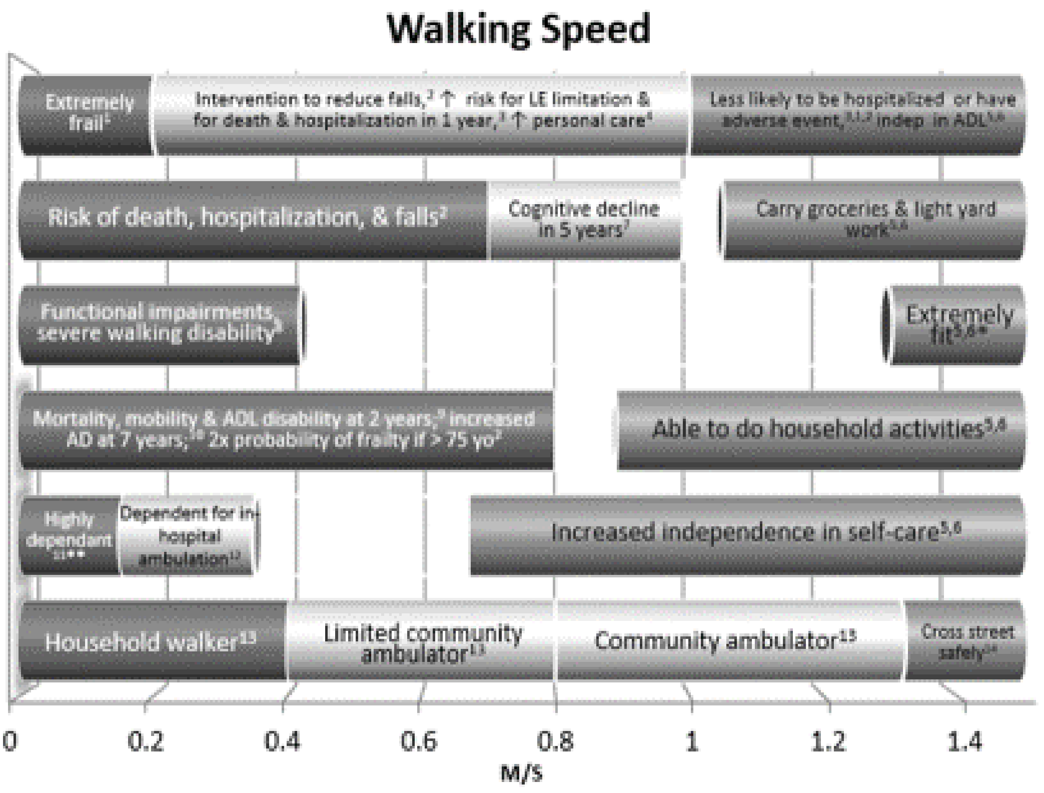

Middleton et al 2015

10 BEAN TRANSFER TEST

General information:

Individual moves 10 beans from table to cup as fast as possible while being timed. The task is performed on the dominant hand and repeated on the non-dominant hand. Only one bean may be moved at one time.

- Start timing when the first bean is collected
- Stop timing when the last bean is placed in the cup
- Documentation should include the time taken to perform task on each side
- Collect one trial on each side.

Set-up:

- Place 10 beans (dried broad beans) in front of the person and place the cup 30 cm away on a table
- Document whether standing or sitting

Patient Instructions:

Starting with the right hand: "I will say ready, set, go. When I say go, using your (R) hand, move the beans, one at a time from the table to the cup as fast as you can."

Repeat using the left hand: "I will say ready, set, go. When I say go, using your (L) hand, move the beans, one at a time from the table to the cup as fast as you can."

6 MINUTE TIMED WALK

General Information:

Individual walks without assistance along a 10 metre walkway for 6 minutes. 180 degree turns are to be taken at the end of each 10 metre length

- start timing when the toes of the leading foot crosses the start
- stop timing at 6 minutes or if the individual cannot continue the test
- assistive devices can be used but should be kept consistent and documented from test to test
- if physical assistance is required to walk, this should not be performed
- Should be performed at fastest speed possible
- documentation should include the metres covered, level of effort required and number of rests required.

Set-up:

- measure and mark a 10-meter walkway
- place a chair at each end of the walkway
- video recording for playback as appropriate

Patient Instructions:

"I will say ready, set, go. When I say go, walk as fast as you can until I say stop. If you need to rest at any time, sit down on the chairs provided. Please note that the stop watch will keep going. Please turn around fully at the end of each length of the walkway."

6 minute walk distances: Steffen et al 2002

Age	Gender (N)	Mean	SD	Normal Range (2SD)
60-69	Male (15)<br>Female (22)	572m (1,877 ft.)<br>538 m (1.765 ft.)	92 m<br>92 m	388-756 m<br>354-722 m
70-79	Male (14)<br>Female (22)	527 m (1.729 ft.)<br>471 m (1,545 ft.)	85 m<br>75 m	357-697 m<br>321-621 m
80-89	Male (8)<br>Female (15)	417 m (1,368 ft.)<br>392 m (1.286 ft.)	73 m<br>85 m	271-563 m<br>222-562 m

PDQ-39 QUESTIONNAIRE

Please complete the following questionnaire by ticking one box for each question. When answering consider the following…..due to having Parkinson's disease, how often during the last month have you……………

Task	Never (0)	Occasionally (1)	Sometimes (2)	Often (3)	Always /unable (4)
Had difficulty doing the leisure activities which you would like to do?					
Had difficulty looking after your home, eg DIY, housework, cooking?					
Had difficulty carrying bags of shopping?					
Had problems walking half a mile?					
Had problems walking 100 yards?					
Had problems getting around the house as easily as you would like?					
Had difficulty getting around in public?					
Needed someone else to accompany you when you went out?					
Felt frightened or worried about falling over in public?					
Been confined to the house more than you would like?					
Had difficulty washing yourself?					
Had difficulty dressing yourself?					
Had problems doing up your shoe laces?					
Had problems writing clearly?					
Had difficulty cutting up your food?					
Had difficulty holding a drink without spilling it?					
Felt depressed?					
Felt isolated and lonely?					
Felt weepy or tearful?					

Felt angry or bitter?					
Felt anxious?					
Felt worried about your future?					
Felt you had to conceal your Parkinson’s from people?					
Avoided situations which involve eating or drinking in public?					
Felt embarrassed in public due to having Parkinson’s disease?					
Felt worried by other people’s reactions to you?					
Had problems with your close personal relationships?					
Lacked support in the ways you needed from your spouse or partner?					
Lacked support in the ways you need from your family or close friends?					
Unexpectedly fallen asleep during the day?					
Had problems with your concentration, eg, when reading or watching TV?					
Felt your memory was bad?					
Had distressing dreams or hallucinations?					
Had difficulty with your speech?					
Felt unable to communicate with people properly?					
Felt ignored by people?					
Had painful muscle cramps or spasms?					
Had aches and pains in your joints or body?					
Felt unpleasantly hot or cold?					
**TOTAL**					

PD WARRIOR

SELF ASSESSMENT PD DISABILITY SCALE

NAME:

Please rate the degree of difficulty you have doing each of these activities in general. If you use a mobility aid, please answer how well you would manage without the aid.

		1 Able to do alone	2 Alone but Minimally difficult	3 Difficult or with help	4 Only able with a lot of help	5 Unable to do at all	Comment	N/A
1	Get out of bed							
2	Get up from an armchair							
3	Walk around the house							
4	Walk outside eg to the local shops							
5	Travel by public transport							
6	Walk up the stairs							
7	Walk down stairs							
8	Wash face and hands							
9	Get into a bath							
10	Get out of a bath							
11	Get dressed							
12	Get undressed							
13	Brush your teeth							
14	Open tins							

		1 Able to do alone	2 Alone but Minimally difficult	3 Difficult or with help	4 Only able with a lot of help	5 Unable to do at all	Comment	N/A
15	Pour milk from a bottle or carton							
16	Make a cup of tea or coffee							
17	Hold a cup and saucer							
18	Wash and dry dishes							
19	Cut food with knife and fork							
20	Pick up an object from the floor							
21	Insert and remove an electric plug							
22	Dial a telephone							
23	Hold and read a newspaper							
24	Write a letter							
25	Turn over in bed							

Comments

SCORE: Standard Tasks

Custom Tasks

Mini-BESTest: Balance Evaluation Systems Test
© 2005-2013 Oregon Health & Science University. All rights reserved.

ANTICIPATORY **SUB SCORE: ___ /6**

1. SIT TO STAND
Instruction: "Cross your arms across your chest. Try not to use your hands unless you must. Do not let your legs lean against the back of the chair when you stand. Please stand up now."
(2) Normal: Comes to stand without use of hands and stabilizes independently.
(1) Moderate: Comes to stand WITH use of hands on first attempt.
(0) Severe: Unable to stand up from chair without assistance, OR needs several attempts with use of hands.

2. RISE TO TOES
Instruction: "Place your feet shoulder width apart. Place your hands on your hips. Try to rise as high as you can onto your toes. I will count out loud to 3 seconds. Try to hold this pose for at least 3 seconds. Look straight ahead. Rise now."
(2) Normal: Stable for 3 s with maximum height.
(1) Moderate: Heels up, but not full range (smaller than when holding hands), OR noticeable instability for 3 s.
(0) Severe: $\leq$ 3 s.

3. STAND ON ONE LEG
Instruction: "Look straight ahead. Keep your hands on your hips. Lift your leg off of the ground behind you without touching or resting your raised leg upon your other standing leg. Stay standing on one leg as long as you can. Look straight ahead. Lift now."

Left: *Time in Seconds Trial 1:_____ Trial 2:_____*
(2) Normal: 20 s.
(1) Moderate: < 20 s.
(0) Severe: Unable.

Right: *Time in Seconds Trial 1:_____ Trial 2:_____*
(2) Normal: 20 s.
(1) Moderate: < 20 s.
(0) Severe: Unable

To score each side separately use the trial with the longest time.
To calculate the sub-score and total score use the side [left or right] with the lowest numerical score [i.e. the worse side].

REACTIVE POSTURAL CONTROL **SUB SCORE: ___ /6**

4. COMPENSATORY STEPPING CORRECTION- FORWARD
Instruction: "Stand with your feet shoulder width apart, arms at your sides. Lean forward against my hands beyond your forward limits. When I let go, do whatever is necessary, including taking a step, to avoid a fall."
(2) Normal: Recovers independently with a single, large step (second realignment step is allowed).
(1) Moderate: More than one step used to recover equilibrium.
(0) Severe: No step, OR would fall if not caught, OR falls spontaneously.

5. COMPENSATORY STEPPING CORRECTION- BACKWARD
Instruction: "Stand with your feet shoulder width apart, arms at your sides. Lean backward against my hands beyond your backward limits. When I let go, do whatever is necessary, including taking a step, to avoid a fall."
(2) Normal: Recovers independently with a single, large step.
(1) Moderate: More than one step used to recover equilibrium.
(0) Severe: No step, OR would fall if not caught, OR falls spontaneously.

6. COMPENSATORY STEPPING CORRECTION- LATERAL
Instruction: "Stand with your feet together, arms down at your sides. Lean into my hand beyond your sideways limit. When I let go, do whatever is necessary, including taking a step, to avoid a fall."

Left
(2) Normal: Recovers independently with 1 step (crossover or lateral OK).
(1) Moderate: Several steps to recover equilibrium.
(0) Severe: Falls, or cannot step.

Right
(2) Normal: Recovers independently with 1 step (crossover or lateral OK).
(1) Moderate: Several steps to recover equilibrium.
(0) Severe: Falls, or cannot step.

Use the side with the lowest score to calculate sub-score and total score.

SENSORY ORIENTATION **SUB SCORE: ___ /6**

7. STANCE (FEET TOGETHER); EYES OPEN, FIRM SURFACE
Instruction: "Place your hands on your hips. Place your feet together until almost touching. Look straight ahead. Be as stable and still as possible, until I say stop."
Time in seconds:________
(2) Normal: 30 s.
(1) Moderate: < 30 s.
(0) Severe: Unable.

8. STANCE (FEET TOGETHER); EYES CLOSED, FOAM SURFACE
Instruction: "Step onto the foam. Place your hands on your hips. Place your feet together until almost touching. Be as stable and still as possible, until I say stop. I will start timing when you close your eyes."

*Time in seconds:*________

(2) Normal: 30 s.
(1) Moderate: < 30 s.
(0) Severe: Unable.

9. INCLINE- EYES CLOSED
Instruction: "Step onto the incline ramp. Please stand on the incline ramp with your toes toward the top. Place your feet shoulder width apart and have your arms down at your sides. I will start timing when you close your eyes."

*Time in seconds:*________

(2) Normal: Stands independently 30 s and aligns with gravity.
(1) Moderate: Stands independently <30 s OR aligns with surface.
(0) Severe: Unable.

DYNAMIC GAIT **SUB SCORE: ____ /10**

10. CHANGE IN GAIT SPEED
Instruction: "Begin walking at your normal speed, when I tell you 'fast', walk as fast as you can. When I say 'slow', walk very slowly."

(2) Normal: Significantly changes walking speed without imbalance.
(1) Moderate: Unable to change walking speed or signs of imbalance.
(0) Severe: Unable to achieve significant change in walking speed AND signs of imbalance.

11. WALK WITH HEAD TURNS – HORIZONTAL
Instruction: "Begin walking at your normal speed, when I say "right", turn your head and look to the right. When I say "left" turn your head and look to the left. Try to keep yourself walking in a straight line."

(2) Normal: performs head turns with no change in gait speed and good balance.
(1) Moderate: performs head turns with reduction in gait speed.
(0) Severe: performs head turns with imbalance.

12. WALK WITH PIVOT TURNS
Instruction: "Begin walking at your normal speed. When I tell you to 'turn and stop', turn as quickly as you can, face the opposite direction, and stop. After the turn, your feet should be close together."

(2) Normal: Turns with feet close FAST (≤ 3 steps) with good balance.
(1) Moderate: Turns with feet close SLOW (≥4 steps) with good balance.
(0) Severe: Cannot turn with feet close at any speed without imbalance.

13. STEP OVER OBSTACLES
Instruction: "Begin walking at your normal speed. When you get to the box, step over it, not around it and keep walking."

(2) Normal: Able to step over box with minimal change of gait speed and with good balance.
(1) Moderate: Steps over box but touches box OR displays cautious behavior by slowing gait.
(0) Severe: Unable to step over box OR steps around box.

14. TIMED UP & GO WITH DUAL TASK ***[3 METER WALK]***
Instruction TUG: "When I say 'Go', stand up from chair, walk at your normal speed across the tape on the floor, turn around, and come back to sit in the chair."
Instruction TUG with Dual Task: "Count backwards by threes starting at ___. When I say 'Go', stand up from chair, walk at your normal speed across the tape on the floor, turn around, and come back to sit in the chair. Continue counting backwards the entire time."

TUG: ________seconds; Dual Task TUG: ________seconds

(2) Normal: No noticeable change in sitting, standing or walking while backward counting when compared to TUG without Dual Task.
(1) Moderate: Dual Task affects either counting OR walking (>10%) when compared to the TUG without Dual Task.
(0) Severe: Stops counting while walking OR stops walking while counting.

When scoring item 14, if subject's gait speed slows more than 10% between the TUG without and with a Dual Task the score should be decreased by a point.

TOTAL SCORE: ____ /28

Mini-BESTest Instructions

Subject Conditions: Subject should be tested with flat-heeled shoes OR shoes and socks off.
Equipment: Temper® foam (also called T-foam™ 4 inches thick, medium density T41 firmness rating), chair without arm rests or wheels, incline ramp, stopwatch, a box (9" height) and a 3 meter distance measured out and marked on the floor with tape [from chair].
Scoring: The test has a maximum score of **28** points from **14 items** that are each scored from 0-2.
"0" indicates the lowest level of function and "2" the highest level of function.
If a subject must use an assistive device for an item, score that item one category lower.
If a subject requires physical assistance to perform an item, score "0" for that item.
For **Item 3** (stand on one leg) and **Item 6** (compensatory stepping-lateral) only include the score for one side (the worse score).
For **Item 3** (stand on one leg) select the best time of the 2 trials [from a given side] for the score.
For **Item 14** (timed up & go with dual task) if a person's gait slows greater than 10% between the TUG without and with a dual task then the score should be decreased by a point.

1. SIT TO STAND	Note the initiation of the movement, and the use of the subject's hands on the seat of the chair, the thighs, or the thrusting of the arms forward.
2. RISE TO TOES	Allow the subject two attempts. Score the best attempt. (If you suspect that subject is using less than full height, ask the subject to rise up while holding the examiners' hands.) Make sure the subject looks at a non-moving target 4-12 feet away.
3. STAND ON ONE LEG	Allow the subject two attempts and record the times. Record the number of seconds the subject can hold up to a maximum of 20 seconds. Stop timing when the subject moves hands off of hips or puts a foot down. Make sure the subject looks at a non-moving target 4-12 feet ahead. Repeat on other side.
4. COMPENSATORY STEPPING CORRECTION-FORWARD	Stand in front of the subject with one hand on each shoulder and ask the subject to lean forward (Make sure there is room for them to step forward). Require the subject to lean until the subject's shoulders and hips are in front of toes. After you feel the subject's body weight in your hands, very suddenly release your support. The test must elicit a step. NOTE: Be prepared to catch subject.
5. COMPENSATORY STEPPING CORRECTION - BACKWARD	Stand behind the subject with one hand on each scapula and ask the subject to lean backward (Make sure there is room for the subject to step backward.) Require the subject to lean until their shoulders and hips are in back of their heels. After you feel the subject's body weight in your hands, very suddenly release your support. Test must elicit a step. NOTE: Be prepared to catch subject.
6. COMPENSATORY STEPPING CORRECTION- LATERAL	Stand to the side of the subject, place one hand on the side of the subject's pelvis, and have the subject lean their whole body into your hands. Require the subject to lean until the midline of the pelvis is over the right (or left) foot and then suddenly release your hold. NOTE: Be prepared to catch subject.
7. STANCE (FEET TOGETHER); EYES OPEN, FIRM SURFACE	Record the time the subject was able to stand with feet together up to a maximum of 30 seconds. Make sure subject looks at a non-moving target 4-12 feet away.
8. STANCE (FEET TOGETHER); EYES CLOSED, FOAM SURFACE	Use medium density Temper® foam, 4 inches thick. Assist subject in stepping onto foam. Record the time the subject was able to stand in each condition to a maximum of 30 seconds. Have the subject step off of the foam between trials. Flip the foam over between each trial to ensure the foam has retained its shape.
9. INCLINE EYES CLOSED	Aid the subject onto the ramp. Once the subject closes eyes, begin timing and record time. Note if there is excessive sway.
10. CHANGE IN SPEED	Allow the subject to take 3-5 steps at normal speed, and then say "fast". After 3-5 fast steps, say "slow". Allow 3-5 slow steps before the subject stops walking.
11. WALK WITH HEAD TURNS-HORIZONTAL	Allow the subject to reach normal speed, and give the commands "right, left" every 3-5 steps. Score if you see a problem in either direction. If subject has severe cervical restrictions allow combined head and trunk movements.
12. WALK WITH PIVOT TURNS	Demonstrate a pivot turn. Once the subject is walking at normal speed, say "turn and stop." Count the number of steps from "turn" until the subject is stable. Imbalance may be indicated by wide stance, extra stepping or trunk motion.
13. STEP OVER OBSTACLES	Place the box (9 inches or 23 cm height) 10 feet away from where the subject will begin walking. Two shoeboxes taped together works well to create this apparatus.
14. TIMED UP & GO WITH DUAL TASK	*Use the TUG time to determine the effects of dual tasking. The subject should walk a 3 meter distance. TUG:* Have the subject sitting with the subject's back against the chair. The subject will be timed from the moment you say "Go" until the subject returns to sitting. Stop timing when the subject's buttocks hit the chair bottom and the subject's back is against the chair. The chair should be firm without arms. *TUG With Dual Task:* While sitting determine how fast and accurately the subject can count backwards by threes starting from a number between 100-90. Then, ask the subject to count from a different number and after a few numbers say "Go". Time the subject from the moment you say "Go" until the subject returns to the sitting position. Score dual task as affecting counting or walking if speed slows (>10%) from TUG and or new signs of imbalance.

http://www.bestest.us/files/7413/6380/7277/MiniBEST_revised_final_3_8_13.pdf

PD WARRIOR

WRITING PRACTICE

PD NMS QUESTIONNAIRE

Name: .... Date: .... Age: ....

Centre ID: Male ☐ Female ☐

NON-MOVEMENT PROBLEMS IN PARKINSON'S

The movement symptoms of Parkinson's are well known. However, other problems can sometimes occur as part of the condition or its treatment. It is important that the doctor knows about these, particularly if they are troublesome for you.

A range of problems is listed below. Please tick the box 'Yes' if you have experienced it **during the past month.** The doctor or nurse may ask you some questions to help decide. If you have **not** experienced the problem in the past month tick the 'No' box. You should answer 'No' even if you have had the problem in the past but not in the past month.

Have you experienced any of the following in the last month?

	Yes	No
1. Dribbling of saliva during the daytime	☐	☐
2. Loss or change in your ability to taste or smell	☐	☐
3. Difficulty swallowing food or drink or problems with choking	☐	☐
4. Vomiting or feelings of sickness (nausea)	☐	☐
5. Constipation (less than 3 bowel movements a week) or having to strain to pass a stool (faeces)	☐	☐
6. Bowel (fecal) incontinence	☐	☐
7. Feeling that your bowel emptying is incomplete after having been to the toilet	☐	☐
8. A sense of urgency to pass urine makes you rush to the toilet	☐	☐
9. Getting up regularly at night to pass urine	☐	☐
10. Unexplained pains (not due to known conditions such as arthritis)	☐	☐
11. Unexplained change in weight (not due to change in diet)	☐	☐
12. Problems remembering things that have happened recently or forgetting to do things	☐	☐
13. Loss of interest in what is happening around you or doing things	☐	☐
14. Seeing or hearing things that you know or are told are not there	☐	☐
15. Difficulty concentrating or staying focussed	☐	☐
16. Feeling sad, 'low' or 'blue'	☐	☐
17. Feeling anxious, frightened or panicky	☐	☐
18. Feeling less interested in sex or more interested in sex	☐	☐
19. Finding it difficult to have sex when you try	☐	☐
20. Feeling light headed, dizzy or weak standing from sitting or lying	☐	☐
21. Falling	☐	☐
22. Finding it difficult to stay awake during activities such as working, driving or eating	☐	☐
23. Difficulty getting to sleep at night or staying asleep at night	☐	☐
24. Intense, vivid dreams or frightening dreams	☐	☐
25. Talking or moving about in your sleep as if you are 'acting' out a dream	☐	☐
26. Unpleasant sensations in your legs at night or while resting, and a feeling that you need to move	☐	☐
27. Swelling of your legs	☐	☐
28. Excessive sweating	☐	☐
29. Double vision	☐	☐
30. Believing things are happening to you that other people say are not true	☐	☐

DUAL TASKING EXAMPLES

- Please give me all the girls/boys names going through the alphabet (a= Annabelle, b= bridgit)
- Name all the Asian countries you can think of
- Objects found in the bathroom, garden shed, kitchen
- Bird species, fish species, insects, dog breeds, cat breeds
- Capital cities in Africa, Europe, South America, etc
- Brands of cars
- Name the chess pieces
- Name all the items you can think of that are blue, green, red, grey etc
- Name all the countries that are largely desert, snowy, tropical etc
- Name all the designer clothes brands
- Name all the flowers you can think of
- Name all the international airlines
- List the last 5 addresses you have lived in
- Count backwards from X in 7s, 3s, etc
- If I say 35, you say ***even***, if I say 64, you say ***odd***
- Name all the opposite pairs you can think of
- Name all the different pizza toppings you can think of
- Name all the different cuisines you can think of
- Name all the Olympic sports you can think of
- Name all the Australian Prime Ministers you can think of
- List all the prime numbers
- List all the professions you can think of
- List all the trades you can think of
- Name all the recipes that use eggs
- Please go through your 3, 6, 7, 9 times tables
- Please spell your name backwards
- When I say red, you say GO, when I say green, you say STOP
- Please name your top 5 favourite movies
- Name all the famous movie stars, sports stars, animals in the zoo

1.

2.

3.

4.

5.
