# Supplementary material for: Functional Outcomes Following a 10-Week PD Warrior Program: A Real-World Clinical Follow-Up of a Neuroplasticity-Driven Exercise Approach
Source: J Clin Med. 2026 Oct 14;15(20):7915. doi: 10.3390/jcm15207915 (PMC13706282; doi:10.3390/jcm15207915)
Supplement: Supplementary file 1 [file jcm-15-07915-s001.zip › Supplementary material S2.pdf]

Outcome Measure Testing Protocols

The **Hoehn & Yahr (H&Y) stage** was the measure recorded and analyzed for disease severity/stage. H&Y staging classifies Parkinson's disease according to the distribution of motor involvement and the presence and severity of postural instability, ranging from Stage 1 (unilateral involvement) through Stage 5 (bedridden unless aided). H&Y stage was determined clinically by the MDS-UPDRS-certified physiotherapist based on the participant's neurological presentation, including the relevant motor examination findings and the H&Y staging criteria.

The **Mini-Balance Evaluation Systems Test (Mini-BESTest)** was administered as a clinical measure of dynamic balance. The assessment consists of 14 items examining four domains of postural control: anticipatory postural adjustments, reactive postural control, sensory orientation, and dynamic gait. Individual items are scored from 0 to 2, with higher scores indicating better balance performance and a maximum total score of 28.

The **Timed Up and Go (TUG)** was used to assess functional mobility. Participants began seated in a chair and, when instructed, stood from the chair, walked three metres, turned, returned to the chair, and sat down. The time required to complete the full sequence was recorded in seconds. Participants completed the test using their typical footwear and, where clinically required, their usual walking aid. As with the other mobility assessments, these conditions were retained to reflect routine clinical practice and the participant's functional mobility.

The **10-Meter Walk Test (10MWT)** was used to assess walking speed. Participants were instructed to walk a designated 14-metre distance as quickly and safely as possible without running, with testers recording the time taken and number of steps required to complete an intermediate 10-metre distance, after the acceleration and before the deceleration stages. Number of steps taken to traverse the 10-metre distance was converted to step length (10 divided by step-length), and time taken to traverse the 10-metre distance was converted to speed (10 divided by time).

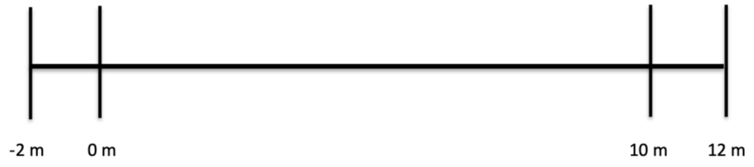

Participants completed the assessment using their typical footwear and any walking aid that they routinely required. Footwear and assistive devices were not standardized because the data were collected during routine clinical care, with the intention of assessing functional mobility under conditions representative of each participant's usual real-world mobility. Therefore, the values reported in this study represent fast walking speed rather than usual or self-selected walking speed. Time to complete the distance was recorded and used to determine walking performance/speed.
